# A Novel Scoring System Based on the Level of HDL-C for Predicting the Prognosis of t-DLBCL Patients: A Single Retrospective Study

**DOI:** 10.1155/2018/2891093

**Published:** 2018-12-30

**Authors:** Jiadai Xu, Zheng Wei, Yian Zhang, Chen Chen, Jing Li, Peng Liu

**Affiliations:** Department of Hematology, Zhongshan Hospital, Fudan University, Shanghai, China

## Abstract

The t-DLBCL patients are generally regarded to experience a poor prognosis. However, there is little consensus to guide optimal management strategies for such patients group. The present study aimed to explore the incidence of transformation and the prognosis factors for t-DLBCL patients, thereby providing insights for clinical choices. We retrospectively investigated 46 patients with diffuse large B-cell lymphomas (DLBCL) associated with an indolent small B-cell non-Hodgkin lymphoma (iNHL) from January 2007 to June 2017 in our department. In multivariate analysis, bone marrow (BM) involvement and low level of high-density lipoprotein cholesterol (HDL-C) were considered as two negatively and independently prognostic factors for overall survival (OS) (BM:* p*=0.007, HR 7.475, 95%CI: 1.744-32.028; HDL-C:* p*=0.032, HR10.037, 95%CI: 1.226-82.162). International Prognostic Index (IPI) risk group was identified as a single independent prognostic factor of progression-free survival (PFS) (*p*=0.048, HR 2.895, 95%CI: 1.010-8.297). A novel prognostic scoring system named BH model (BH stands for the intertwined initials of BM situation and the level of HDL-C) was further developed to stratify these patients into two risk groups, which performed well. Combining the BH scoring model and IPI scoring system could better predict the outcomes of these patients.

## 1. Introduction

Globally, the annual incidence rate of non-Hodgkin lymphoma (NHL) from 2007 to 2011 was 19.7/100,000 [1]. NHL incidence has continued to increase among non-HIV affected population from 1990-2009, which significantly develops faster than that of any other malignancies except lung cancer, melanoma, and prostate cancer [2]. NHL is divided into B, T, and NK cell lymphomas, and the B- and T-cell lymphomas are further subdivided into precursor versus mature subtypes according to WHO classification. B-cell NHL is clinically stratified as indolent (e.g., follicular, marginal zone, and small lymphocytic) versus aggressive (e.g., diffuse large B-cell lymphomas (DLBCL), mantle cell, and Burkitt) subtypes. DLBCL consists of heterogeneous subtypes that can be de novo or transformed from indolent lymphoma [3].

According to the Wintrobe's Clinical Hematology, discordant lymphoma is encountered when DLBCL transforms from or coexists with indolent lymphoma in the bone marrow or lymph node. In most cases, this represents that an aggressive component has transformed from a preexisting indolent B-cell clone. Typical clinical manifestations of transformation include rapidly enlarged lymph nodes, unexplained B-type symptoms, or significantly elevated serum calcium level or serum lactate dehydrogenase (LDH) level [4]. Here, DLBCL associated with a low-grade small B-cell NHL is abbreviated as t-DLBCL (DLBCL associated with transformation) below. According to the survey from Hervé Ghesquières et al., although there is no significant difference in the overall survival (OS) between t-DLBCL and de novo DLBCL, lower rates of complete response (60%* versus *79%) and decreased 5-year freedom from progression (FFP) rate (33%* versus *57%) were found in t-DLBCL [5]. Rituximab-based immunochemotherapy has advanced in recent years; however, t-DLBCL still remains a challenge in the treatment of DLBCL. Generally, t-DLBCL patients are usually excluded from clinical trials and are more likely to experience refractoriness or relapse, leading to unsatisfactory clinical outcomes [6, 7]. In the study of Mary Gleeson et al. [8], presence of B symptoms and International Prognostic Index (IPI) risk at transformation were significant independent prognostic factors for OS of DLBCL associated with follicular lymphoma (FL). Besides, in the study of Hervé Ghesquières et al. [5], IPI, age-adjusted IPI score, treatment by high-dose therapy, Eastern Cooperative Oncology Group (ECOG) status, and complete response to first-line therapy were variables that predict OS and FFP for t-DLBCL patients. However, retrospective analysis in Chinese ethnicity that specifically targeted on t-DLBCL patients were relatively rare. Therefore, we aimed to explore the incidence of transformation and the prognosis factors for t-DLBCL patients, thereby providing insights for clinical choices, especially for Chinese patients.

## 2. Patients and Methods

### 2.1. Patients

DLBCL cases with a period of over 10 years (from January 2007 to June 2017) were searched from histopathology electronic database in our hospital. Electronic records of these DLBCL cases were reviewed to identify 46 t-DLBCL patients. Consistent with previous studies, transformation was diagnosed when the proportion of histological large B-cells exceeded 20% of the small B-cell population and when the obvious mitotic phase was observed [9, 10]. The study was approved by the ethics committee in our hospital and strictly abided by the Declaration of Helsinki.

### 2.2. Pathological and Clinical Data

A panel of monoclonal antibodies including anti-MUM1, CD3, CD5, CD10, CD20, CD21, CD23, CD43, CD79a, LCA, Bcl-2, Bcl-6, and Ki-67 were used in the pathological diagnosis by immunohistochemistry. The following tests were performed in each newly diagnosed t-DLBCL patient, including blood counts, urine and stool tests, liver and kidney function, serum albumin, immunoglobulin, C-reactive protein (CRP), protein electrophoresis, serum lipid level, LDH, *β*2-microglobulin (*β*2-MG), systematic CT or PET-CT scan, and bone marrow (BM) aspiration. Hans-algorithms classification, general status, Ann Arbor stage, and IPI score of each patient were evaluated.

### 2.3. Treatment

Among the 46 t-DLBCL patients, 43 of them were enrolled in the formal therapy, while other three patients gave up treatment with personal reasons. Rituximab, cyclophosphamide, doxorubicin, vincristine, and prednisone, referred as R-CHOP, were the frontline regimen for DLBCL according to the 2017 National Comprehensive Cancer Network (NCCN) guideline. R-CHOP chemotherapy was prescribed in the treatment of t-DLBCL patients without contraindications, such as severe infection of hepatitis virus B, patient unwillingness, or previous application of R-CHOP. The other patients were administrated appropriate regimens according to their individual conditions, including a combination of cyclophosphamide, doxorubicin, vincristine, and prednisone based (CHOP based), ifosfamide, carboplatin and etoposide (ICE) or gemcitabine, cisplatin, and dexamethasone (GDP).

### 2.4. End Points Assessment

Overall survival (OS) was defined as the period from the diagnostic date of transformation, regardless of whether the patients had previously suffered prehistory of indolent NHL (iNHL) or not, to death or the last follow-up. Progression-free survival (PFS) was defined as the period from diagnosis of t-DLBCL to disease progression, death or last follow-up. Complete remission (CR) was defined as complete disappearance of all clinical manifestations or radiologic lymphoma lesions, which was normally evaluated by positron emission tomography (PET) scan or computed tomography (CT). Partial remission (PR) was defined as at least a 50% regression of measurable disease without new lesions. Overall response rate (ORR) was defined as the rate of CR rate plus PR rate.

### 2.5. Statistical Analysis

One-way analysis of variance (one-way ANOVA) and *χ*2 test (or Fisher's exact test) were used to compare continuous and categorical variables, respectively. Survival analysis was carried out with Kaplan-Meier method (log-rank test) and stepwise Cox regression analysis. As an exploratory toll, stepwise Cox regression analysis was used to develop a novel prognostic scoring system. Time-dependent receiver operating characteristic curve (time-dependent ROC) was used to evaluate the discriminatory ability of this model. The nomogram and time-dependent ROC were established with R and Empower Stats software. Other statistical analyses were performed using the IBM SPSS Statistics 23.00. Two-sided p value ≤ 0.05 was considered statistically significant. Missing variable values were excluded from the complete questionnaire.

## 3. Results

### 3.1. Baseline Characteristics of the 46 t-DLBCL Patients

There were totally 46 newly diagnosed t-DLBCL patients in our department from January 1^st^, 2007, to June 30^th^, 2017. 28 patients had their histological results of lymph node biopsy showing coexistence of large and small malignant B-cells at diagnosis (Table 1). These patients did not have clear histories of pure iNHL. For the remaining 18 patients, who had clear iNHL histories, had large malignant B-cells found in histological results during the course of iNHL. Their average transformation period was 37.73 months (from 1.97 months to 140.00 months). Baseline clinical characteristics of 46 t-DLBCL patients were summarized in Table 1. Among these patients, 25 of them were associated with FL (hereinafter referred to as FL/DLCBL group), 15 were associated with marginal zone lymphomas (MZL) (referred to as MZL/DLBCL group), and the remaining 6 were grouped into others/DLBCL. In the MZL/DLBCL group, 14 of them were MALToma patients (referred to as MALT/DLBCL group) and the other one was nodal marginal zone B-cell lymphoma (NMZL) patient. Among the 14 MALToma patients, half of them were developed from gastric tissue. In the others/DLBCL group, three of them were unclassified B-cell NHL patients, two were small lymphocytic lymphoma (SLL) patients, and one was B-cell chronic lymphoproliferative disorders (B-CLPD) patient.

Differences in the clinical characteristics between the three morphologic groups were also listed in Table 1. Wholly transformed t-DLBCL was defined as complete transformation of large B-cells without any remaining features of small B-cells proliferation in nodal biopsies. Partially coexisted t-DLBCL was defined as a mix of large B-cell population with small B-cell proliferation in nodal biopsies. We found that the MZL/DLBCL group had the lowest proportion (13.3%) of wholly transformed t-DLBCL patients. The FL/DLBCL group had a middle proportion (32.0%) while the other/DLBCL group had the highest one (83.3%). The difference was statistically significant (*p=*0.008). Moreover, a total of 25 patients (32.6%) suffered BM involvement in our cohort. The others/DLBCL group was the most susceptible to BM involvement (50.0%), MZL/DLBCL group showed the lowest proportion of being involved in BM (6.7%), and FL/DLBCL group was in the middle of two groups (44.0%) (*p*=0.032).

We further summarized the pathologic characteristics of the 46 t-DLBCL patients. Expressions of Bcl-2 (21 of 23 tested), Bcl-6 (20 of 23 tested), CD20 (24 of 24 tested), CD23 (17 of 17 tested), CD43 (3 of 3 tested), CD79a (24 of 24 tested), and LCA (8 of 8 tested) were detected in patients from FL/DLBCL group. Expressions of Mum1 (9 of 11 tested), Bcl-6 (11 of 13 tested), CD20 (14 of 14 tested), CD43 (2 of 2 tested), and CD79a (14 of 14 tested) were detected in patients from MZL/DLBCL group. Expressions of CD20 (6 of 6 tested), CD43 (2 of 2 tested), CD79a (5 of 5 tested), and LCA (3 of 3 tested) were detected in patients from others/DLBCL group.

### 3.2. Response to Treatment and the Survival of the 43 t-DLBCL Patients Who Received Therapy

**Figure 1:**
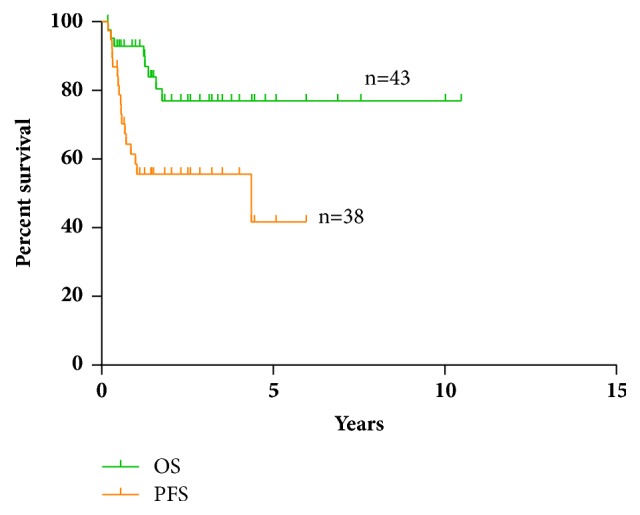
The OS and PFS of 43 t-DLBCL patients.

Among 43 patients who received treatment, 32 patients (74.4%) were treated with immunochemotherapy based on Rituximab, and 38 patients had regular follow-ups. 10.5% patients (4 of 38 cases) achieved CR, including 3 FL/DLBCL patients and 1 MZL/DLBCL patient. Besides, 65.8% patients (25 of 38 cases) achieved PR, including 11 FL/DLBCL patients, 10 MZL/DLBCL patients, and 4 others/DLBCL patients. Based on the results above, ORR of all enrolled patients was 76.3%. There were no significant differences of CR rate and PR rate among the three groups. Detailed data were shown in Table 2. During the follow-up period, there were 8 death cases and 10 censored cases. The median follow-up period was 3.17 years (0.51 years-10.61 years), whereas the median PFS and OS were 4.36 years and not reached, respectively (Figure 1). The estimated 1-year and 5-year survival rates were 93.0% and 77.0%, respectively.

### 3.3. Prognostic Factors for OS and PFS of the t-DLBCL Patients

**Figure 2:**
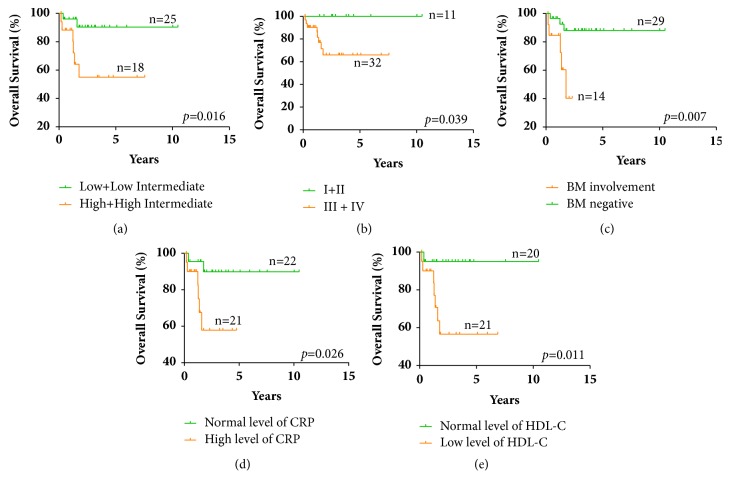
Significant prognostic factors for OS of t-DLBCL patients in univariant analysis. (a) IPI risk groups. (b) Ann Arbor stages. (c) BM situation. (d) CRP level at diagnosis. (e) HDL-C level at diagnosis.

**Figure 3:**
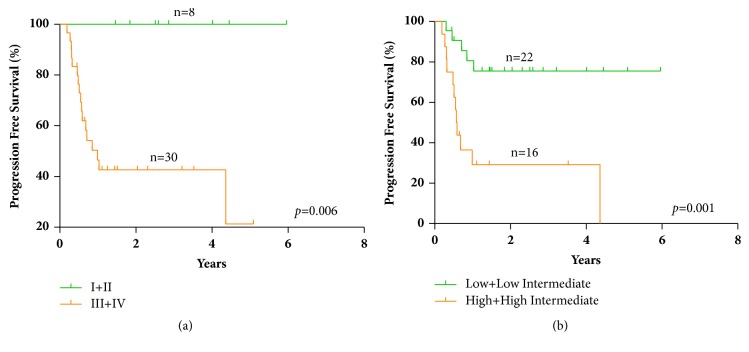
Significant prognostic factors for PFS of t-DLBCL patients in univariant analysis. (a) Ann Arbor stages. (b) IPI risk groups.

In order to identify the clinical prognostic factors for t-DLBCL patients, we performed univariate survival analysis by Kaplan-Meier analysis (log-rank test) and multivariate analysis by Cox regression analysis in the 43 treatment-received patients. In univariate analysis, we identified that high-risk groups of IPI (high and high intermediate) (Figure 2(a),* p*=0.016), high Ann Arbor stages (III+IV) (Figure 2(b),* p*=0.039), BM involvement (Figure 2(c),* p*=0.007), high level of CRP at diagnosis (Figure 2(d),* p*=0.026), and low level of high-density lipoprotein cholesterol (HDL-C) at diagnosis (Figure 2(e), p=0.011) were 5 significant adverse factors for OS. No significant difference was found between germinal center B-cell-like (GCB) and non-GCB type according to Hans-algorithms classification (*p*=0.619). As for PFS, high Ann Arbor stages (III+IV) (Figure 3(a), p=0.006) and high-risk groups of IPI (high and high intermediate) (Figure 3(b),* p*=0.001) were identified as significant adverse prognostic factors.

According to the results from univariate analysis, we constructed multivariate analysis in stepwise Cox regression to evaluate the prognostic significance of these covariates. Two independent prognostic factors, BM situation (*p*=0.007, HR 7.475, 95%CI: 1.744-32.028) and HDL-C level at diagnosis (*p*=0.032, HR10.037, 95%CI: 1.226-82.162), were identified for OS (Table 3). IPI risk groups (*p*=0.048, HR 2.895, 95%CI: 1.010-8.297) were identified as the single independent prognostic factor for PFS.

### 3.4. A Prognostic Scoring System for Predicting the Prognosis of t-DLBCL Patients

**Figure 4:**
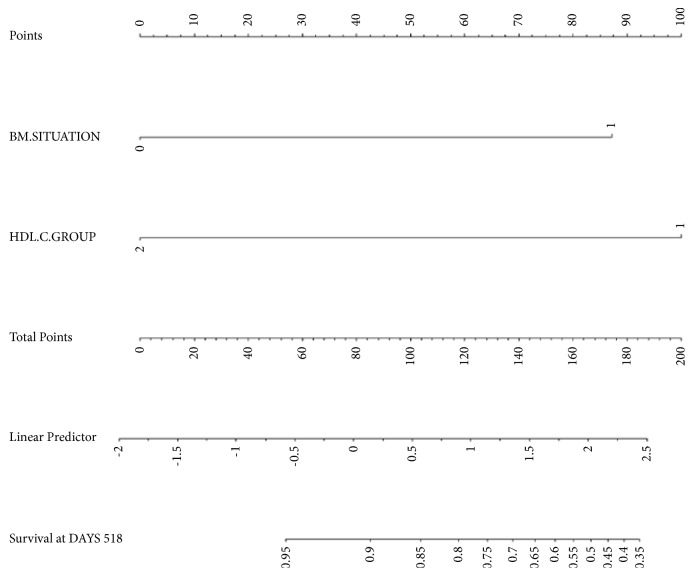
The nomogram, including BM situation and HDL-C level at diagnosis, based on the results from the multivariate Cox regression analysis.

Based on the results from the multivariate analysis, a nomogram was developed (Figure 4). In the nomogram, the situation of the two variables is located on each variable axis. To determine the points of each variable value, the “Points” line is drawn upwards. The sum of these values is located on the “total points” axis, and finally the survival axis was to determine the OS likelihood of these patients.

As the regression coefficient (*β*) from the multivariate model of BM situation was 2.012 (approximately equal to 2.0) and the HDL-C level was -2.306 (approximately equal to 2.3), we further developed an integral model that may predict the prognosis of t-DLBCL patients, namely, BH scoring system (BH stands for the intertwined initials of BM situation and HDL-C) (Table 4). The sum of the above two indicators' score is the prognostic index of t-DLBCL patients.

**Figure 5:**
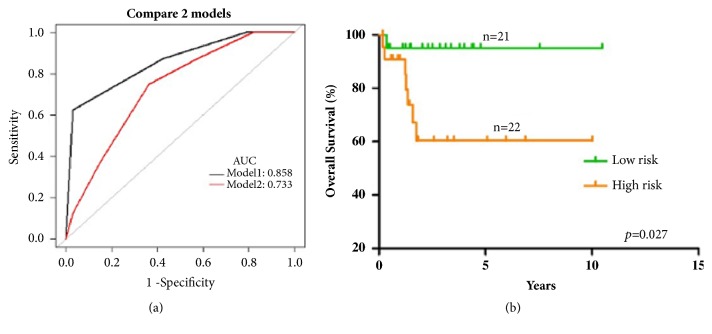
The performance and effectiveness of our BH scoring model. (a) Time-dependent ROC between BH scoring model and the acceptable IPI scoring system. Model 1 (black line) = BH scoring model; Model 2 (red line) = IPI scoring system. (b) The OS between low-risk group and high-risk group, stratified according to the BH scoring model, turned out to be statistically significantly different.

We further compared the performance and effectiveness between our BH scoring model and the acceptable IPI scoring system by time-dependent ROC (Figure 5(a)). For BH scoring model, the area under curve (AUC) was 0.858 (95% CI 0.589–0.961). The cut-off value was -0.131 with a sensitivity of 62.500% and a specificity of 96.970%. For IPI scoring system, the AUC was lower, which was 0.733 (95% CI 0.562–0.921). The cut-off value was -1.397 with a sensitivity of 75.000% and a specificity of 65.850%.

Then we stratified t-DLBCL patients into two risk groups according to the BH scoring system, in which 0-2.0 points stood for low-risk group and 2.3-4.3 points stood for high-risk group. The OS (Figure 5(b),* p*=0.027) between the two risk groups was statistically significantly different.

## 4. Discussion

This analysis was carried out in an unselected cohort of t-DLBCL patients based on nearly 10 years of disease course at a single hematological center. This cohort included 46 patients regardless of gender, profession, economic conditions, or other socioeconomic factors. In the present study, we confirmed that BM situation and HDL-C level at diagnosis were independent factors that influenced OS of t-DLBCL patients. More importantly, our BH scoring system, a novel prognostic index score system, stratifies t-DLBCL patients into two risk groups, which performed well in our research cohort. To the best of our knowledge, this is the first time that HDL-C has been found to be an independent prognostic factor for t-DLBCL patients and decreased HDL-C performed as an early indicator of negative outcome.

Limitations of our study should be addressed to result interpretation. First, this study was carried out retrospectively with a relatively small sample size limited in Chinese patients, which may have potential bias. Second, cytogenetic abnormalities (such as chromosomal translocation or deletion) and gene mutation data were not available until 2012 in our department. So unfortunately, the analysis for double and triple-hit t-DLBCL was unable to be done. Third, according to different individual conditions, treatment plan cannot be completely unified. Fourth, the BH model was based on a single-center retrospective cohort study, which has not been validated in other populations. To confirm the prognostic value of this model on t-DLBCL patients, prospective studies and multicenter studies should be proposed in the future.

What can be learnt from our presented results? Firstly, according to previous studies, HDL-C is associated with sepsis, malignancy, and death. Low concentrations of HDL-C have been reported in patients with hematological malignancies, including DLBCL [11, 12]. In Komiya I's study, in patients with malignant lymphomas and adult T-cell leukemia-lymphoma, cytokine-induced decreased level of HDL-C also performed independent prognostic significance [13]. The underline pathophysiology of this phenomenon still remains unclear. We hypothesize that hematological malignancy may be thought of as an acute phase reaction with hypercytokinaemia, which may influence the lipid metabolism enzymes and lead to lipid derangement. Secondly, BM involvement is clinically recognized as a signature of advanced stage and contributes to higher IPI scores. In our present study, BM involvement adversely impacted OS of t-DLBCL patients, which further emphasize the worse outcome for BM involvement patients.

For patients with previous history of indolent small B-cell NHL, transformation is usually a precipitous event. These patients may suffer from rapidly progressive symptoms within a short period of time. For iNHL patients coexisted with DLBCL when first diagnosed, their clinical performance is commonly more serious than that of pure iNHL patients. Previous studies were generally limited to single clinical case reports or focused on aggressive lymphomas with presence of morphologic small cells in the BM. Limited evidences have greatly restricted our understanding of these groups of patients. Moreover, minor consensus has been achieved to guide optimal management strategies for such patients group. In the present study, comparing with the acknowledged IPI risk groups, the AUC of our BH scoring model was higher, suggesting that it performs better in predicting the prognosis of these patients. The sensitivities between IPI and BH scoring were 75.00% versus 62.50% and the specificities were 65.85% versus 96.97%, which meant that IPI scores had higher sensitivity while BH scores had higher specificity. Therefore, we may combine the two scoring systems together to better predict the final outcomes of t-DLBCL patients. These results may provide insights for personalized clinical decisions. More rigorous treatment regimen and prolonged duration of treatment could be assigned to patients with high risk of poor prognosis. In contrast, patients with low risk could avoid receiving potentially intense toxic therapy unnecessarily. However, it is important to note that future studies are warranted to validate this scoring system.

Finally, we would like to compare the incidence rate between our data and previous studies. Based on current experiences, the risk for FL transformation to DLBCL is about 20% within 8 years [14] and the median time to transformation was 2.5 years [15]. In our study, FL/DLBCL patients accounted for more than half of t-DLBCL patients (54.3%), and the incidence rate was 18.80% within almost 10 years, which was lower than previous studies. Besides, the most common form of histological transformation for MZL is DLBCL [16, 17]. The transformation rate has been reported as 11%-14% for MZL patients, with a median time to transformation of 13 months after the initial diagnosis [18]. In Meyer's study of 197 MZL patients [19], among their transformed MZL cases, 61% were MALT, 26% were nodal MZL, and 13% were SMZL. In our study, 15 MZL/DLBCL patients accounted for 30.4% of overall t-DLBCL patients and among them, 14 (93%) were MALT/DLBCL patients, which presented a quite different composition. These results may have far-reaching consequences for the interpretation and prediction of clinical courses for Chinese t-DLBCL patients.

## 5. Conclusions

In conclusion, in t-DLBCL patients, BM involvement and low level of HDL-C were considered as two negatively and independently prognostic factors for OS. Moreover, IPI risk group is an independent prognostic factor for PFS. Combining our novel BH scoring model and IPI scoring system could better predict the outcomes of these patients.

## Figures and Tables

**Table 1:** Baseline characteristics of 46 t-DLBCL*∗* patients.

**Characteristics, n (**%**)**	**Total patients**		**FL**_ _^**∗**^ / **DLBCL**	**MZL**_ _^**∗**^ /**DLBCL**	**Others/** **DLBCL**	**P value**
	46 (100.0)	NA	25 (54.3)	15 (32.6)	6 (13.0)	
**Age (median, range)**	56, 33-74	0	58, 33-74	53, 39-73	56, 42-70	NS
< 60 years	29 (63.0)		15 (60.0)	9 (60.0)	5 (83.3)	NS
≥60 years	17 (37.0)		10 (40.0)	6 (40.0)	1 (16.7)	
**Gender**		0				
Male	25 (54.3)		14 (56.0)	8 (53.3)	3 (50.0)	NS
Female	21 (45.7)		11 (44.0)	7 (46.7)	3 (50.0)	
**Coexisted with DLBCL ** **at diagnosis**	28 (60.9)	0	14 (56.0)	11 (73.3)	3 (50.0)	NS
**Association with DLBCL ** **in morphologic features**		0				
Wholly transformed	15 (32.6)		8 (32.0)	2 (13.3)	5 (83.3)	**0.008**
Partially co-existed	31 (67.4)		17 (68.0)	13 (86.7)	1 (16.7)	
**Ann Arbor stage**		0				
I-II	11 (24.0)		4 (16.0)	7 (46.7)	0 (0.0)	NS
III	6 (13.0)		3 (12.0)	2 (13.3)	1 (16.7)	
IV	29 (63.0)		18 (72.0)	6 (40.0)	5 (83.3)	
**B symptoms**	24 (52.2)	0	15 (60.0)	6 (40.0)	3 (50.0)	NS
**BM**_ _^**∗**^ ** involvement**	25 (32.6)	0	11 (44.0)	1 (6.7)	3 (50.0)	**0.032**
**IPI**_ _^**∗**^ ** risk group**		0				
Low	16 (34.8)		5 (20.0)	10 (66.7)	1 (16.7)	NS
Low intermediate	10 (21.7)		7 (28.0)	1 (6.7)	2 (33.3)	
High intermediate	10 (21.7)		7 (28.0)	2 (13.3)	1 (16.7)	
High	10 (21.7)		6 (24.0)	2 (13.3)	2 (33.3)	
**Elevated LDH** **∗** **, > 245 U/L**	17 (37.0)	0	11 (44.0)	3 (20.0)	3 (50.0)	NS
**Elevated ** **β** **2 MG, > 2.2 mg/L**	29 (63.0)	0	19 (76.0)	7 (46.7)	3 (50.0)	NS
**Elevated CRP**_ _^**∗**^ **, > 3.0 mg/L**	23 (50.0)	0	13 (52.0)	5 (33.3)	5 (83.3)	NS
**Declined HDL-C**, **≤1.04 mmol/L**	22 (51.2)	3	11 (45.8)	9 (64.3)	3 (60.0)	NS

**DLBCL**, diffuse large B cell lymphoma; **FL**, follicular lymphoma; **MZL**, marginal zone lymphoma; **NA**, not available; **NS**, not significant; **BM**, bone marrow;** IPI**, International Prognostic Index; **LDH**, lactate dehydrogenase; **β2 MG**, *β*2 microglobulin;** CRP**, C-reactive protein; **HDL-C**, high density lipoprotein cholesterol.

**Table 2:** Response to treatment of the t-DLBCL patients.

**Treatment/Response, n (**%**)**	**Total patients**	**F** **L** ^**∗**^/**DLBCL***∗*	**M** **Z** **L** ^**∗**^/**DLBCL**	**Others/** **DLBCL**	**P value**
	N=46	N=25	N=15	N=6	
**Not treated**	3	1	2	0	
**Induction therapy**	43	24	13	6	
R^*∗*^ based	32 (74.4)	17 (70.8)	11 (84.6)	4 (66.7)	
Chemotherapy without R	10 (23.3)	6 (25.0)	2 (15.4)	2 (33.3)	NS
Radiotherapy only	1 (2.3)	1 (4.2)	0 (0.0)	0 (0.0)	
**Response to initial treatment**					
CR^*∗*^	4 (10.5)	3 (15.0)	1 (8.3)	0 (0.0)	NS
PR^*∗*^	25 (65.8)	11 (55.0)	10 (83.3)	4 (66.7)	NS
OR^*∗*^	29 (76.3)	14 (70.0)	11 (91.7)	4 (66.7)	NS
Failure^*∗*^	9 (23.7)	6 (30.0)	1 (8.3)	2 (33.3)	NS
NA^*∗*^	5	4	1	0	
**Survival**					
Estimated median OS^*∗*^, years	NR	NR	NR	1.595^#^	
Estimated median PFS^*∗*^, years	4.364	4.301	5.950	1.020^&^	

^*∗*^R, rituximab; OS, overall survival; PFS, progression free survival; CR, complete remission; PR, partial remission; OR, overall response; NA, not available; NR, not reached; NS, not significant.

^#^Details in the OS of the 6 patients: (1) 1.114 years, alive; (2) 0.194 years, dead; (3) 1.592 years, dead; (4) 4.772 years, alive; (5) 1.444 years, alive; (6) 0.372 years, dead.

^&^Details in the PFS of the 6 patients: (1) 1.114 years, PR; (2) 0.194 years, dead; (3) 1.028 years, PD; (4) 0.675 years, PD; (5) 1.444 years, PR; (6) 0.303 years, PD.

**Table 3:** Multivariable analysis of the association between clinical variables and OS for all patients with t-DLBCL.

**Step**	**Variable**		**OS**		
		**B**	**HR**	**95**%** CI**	***p***
Step 1	BM situation	1.835	6.264	1.464-26.808	0.013
Step 2	BM situation	2.012	7.475	1.744-32.028	**0.007**
	HDL-C level group	2.306	10.037	1.226-82.162	**0.032**

B, regression coefficient; CI, confidence interval; HR, hazard ratio.

Note: IPI risk groups (*p*=0.511), Ann Arbor stage groups (*p*=0.234), and CRP level group (*p*=0.142) were variables that were not entered into the equation.

**Table 4:** BH scoring system for t-DLBCL patients.

**Factors**	**0**	**2**	**2.3**
**BM situation**	Not involvement	Involvement	/
**HDL-C at diagnosis**	Normal	/	Decreased

0-2.0 points = low risk; 2.3-4.3 points = high risk.

## Data Availability

The data used to support the findings of this study are available from the corresponding author upon request.
